# Noninvasive prenatal testing detects microdeletion abnormalities of fetal chromosome 15

**DOI:** 10.1002/jcla.22911

**Published:** 2019-05-15

**Authors:** Lianli Yin, Yinghua Tang, Qing Lu, Mingfang Shi, Aiping Pan, Danyun Chen

**Affiliations:** ^1^ Department of Clinical Laboratory Nanning Second People's Hospital, The Third Affiliated Hospital of Guangxi Medical University Nanning China; ^2^ Department of Clinical Laboratory Guangxi Hospital Of Traditional Chinese Medicine, The First Affiliated Hospital of Guangxi University of Chinese Medicine Nanning China; ^3^ Department of genetic counseling Nanning Second People's Hospital, The Third Affiliated Hospital of Guangxi Medical University Nanning China

**Keywords:** chromosome, copy number variation, deletion, noninvasive prenatal testing

## Abstract

**Objective:**

Noninvasive prenatal testing (NIPT) is widely used in clinical detection of fetal autosomal duplications or deletions. The aim of this study was to investigate the clinical application of NIPT for detection of chromosomal microdeletions.

**Methods:**

Microdeletions of about 5 Mb in the long arm of chromosome 15 (q11.2‐q12) were detected by NIPT and were confirmed by karyotype analysis and copy number variation (CNV) analysis based on high‐throughput sequencing technology.

**Results:**

The CNV results of prenatal diagnosis showed that there were approximately 4.96 Mb of microdeletions in 15q11.2‐q13.1, which was consistent with the NIPT results. The karyotype analysis showed no abnormalities.

**Conclusion:**

In this study, the microdeletion fragment of fetal chromosome 15 was successfully detected and diagnosed using NIPT. This suggests that NIPT is an efficient method to gain genetic information about chromosomal abnormalities.

## 1 INTRODUCTION

Birth defects comprise one of the three major categories of diseases that endanger human health.^1^ Fetal chromosomal abnormality is one of the most important causes of neonatal birth defects. The most common type of abnormality is chromosomal aneuploidy, which includes trisomy 21 (Down's syndrome), trisomy 13 (Patau syndrome), trisomy 18 (Edward's syndrome), and sex chromosome aneuploidy. Noninvasive prenatal testing (NIPT) is widely used in the clinical detection of common fetal aneuploidy due to its high specificity and sensitivity^2^, ^3^, ^4^; however, reports on chromosomal deletions are rare.^5^ In our clinical prenatal screening, we found a case of a mid‐pregnancy patient with an abnormal chromosome 15 deletion. The NIPT results showed a 5‐Mb microdeletion on chromosome 15. Karyotype analysis and CNV test were used to confirm the clinical value of NIPT in chromosome microdeletion.

## 2 CASE INTRODUCTION

A 38‐year‐old pregnant woman, pregnancy 2, parturition 1, gestational age 18 weeks, was sent to the Third Affiliated Hospital of Guangxi Medical University (Nanning, China). The woman was 161 cm tall and weighed 67 kg, and fetal developmental mileage was normal. Ultrasonography at 17 weeks of gestational age had revealed a low placenta and placental maturity level 0. Since pregnant women who are older mothers do not have routine serological screening, NIPT was selected to screen for fetal chromosomal abnormalities. The NIPT results showed that there was a microdeletion of about 5 Mb in the long arm of chromosome 15 at q11.2‐q12. Amniotic fluid was taken for prenatal diagnosis, including karyotype and copy number variation (CNV). The CNV results showed that there were about 4.96 Mb of microdeletions in 15q11.2‐q13.1 (23.62‐28.58), while the karyotype analysis showed no significant abnormalities. The prediagnosis CNV was consistent with the NIPT results.

## 3 MATERIALS AND METHODS

### 3.1 Noninvasive prenatal testing analysis

Operating according to standard procedures (CapitalBio, China), 5 ml of peripheral blood was collected from the pregnant woman and processed within 8 hours. After two low‐temperature centrifugations, the obtained plasma was transferred to a new 2.0 ml‐centrifuge tube and stored at −80°C. The frozen plasma was later reconstituted at room temperature and thoroughly mixed for use. After the DNA extraction, sequencing library preparation, sequencing reaction, enrichment, sequencing, and other processes, the data were analyzed to obtain the Z value. Sequencing was performed using an Ion Proton Sequencing System (Life Technologies). For the test results, Z ≥ 4 was judged as high‐risk; 1.96 < Z < 4 was critical, and amniocentesis was used for a clear diagnosis; and Z ≤ 1.96 was low‐risk and allowed to proceed with the routine pregnancy check process.

### 3.2 Chromosome karyotype analysis

Following the principle of informed and voluntary, the NIPT results suggest that amniocentesis should be performed for high‐risk pregnant women, whose condition was judged to be critical, to verify the fetal karyotype analysis. For this case, the amniocentesis was performed under the guidance of ultrasound in the pregnant woman, and 15‐20 ml of amniotic fluid was taken. The karyotype analysis of fetal amniotic fluid exfoliated cells was performed by visual identification.

### 3.3 CNV analysis based on high‐throughput sequencing technology

Operating according to standard procedures (CapitalBio, China), 10 ml fetal amniotic fluid or 2 ml of uncultured peripheral blood samples were collected, and DNA extraction, sequencing library preparation, sequencing, and other processes were performed as described. Sequencing was performed using an Ion Proton Sequencing System (Life Technologies). The results were analyzed using the chromosome Analysis software. Analyze genotypes non‐type correlations and interpret the data through public databases (decipher, ISCA, omim, Clinva, ucsc, ncbi).

## 4 RESULTS

### 4.1 Noninvasive prenatal testing

**Figure 1:**
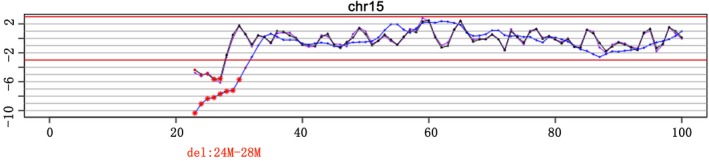
A NIPT study of maternal plasma showing a Z‐score of 2.315 for fetal chromosome 15 and a deletion of approximately 5 Mb from the 24 Mb‐28 Mb region

Noninvasive prenatal testing results gave a Z‐score for chromosome 15 of 2.315 and showed that there was about a deletion of 5 Mb at 24 Mb‐28 Mb (Figure 1). Because these scores indicated that chromosome 15 had a deletion of fetal DNA fragments, the NIPT results were verified with CNV.

### 4.2 Copy number variation analysis

**Figure 2:**
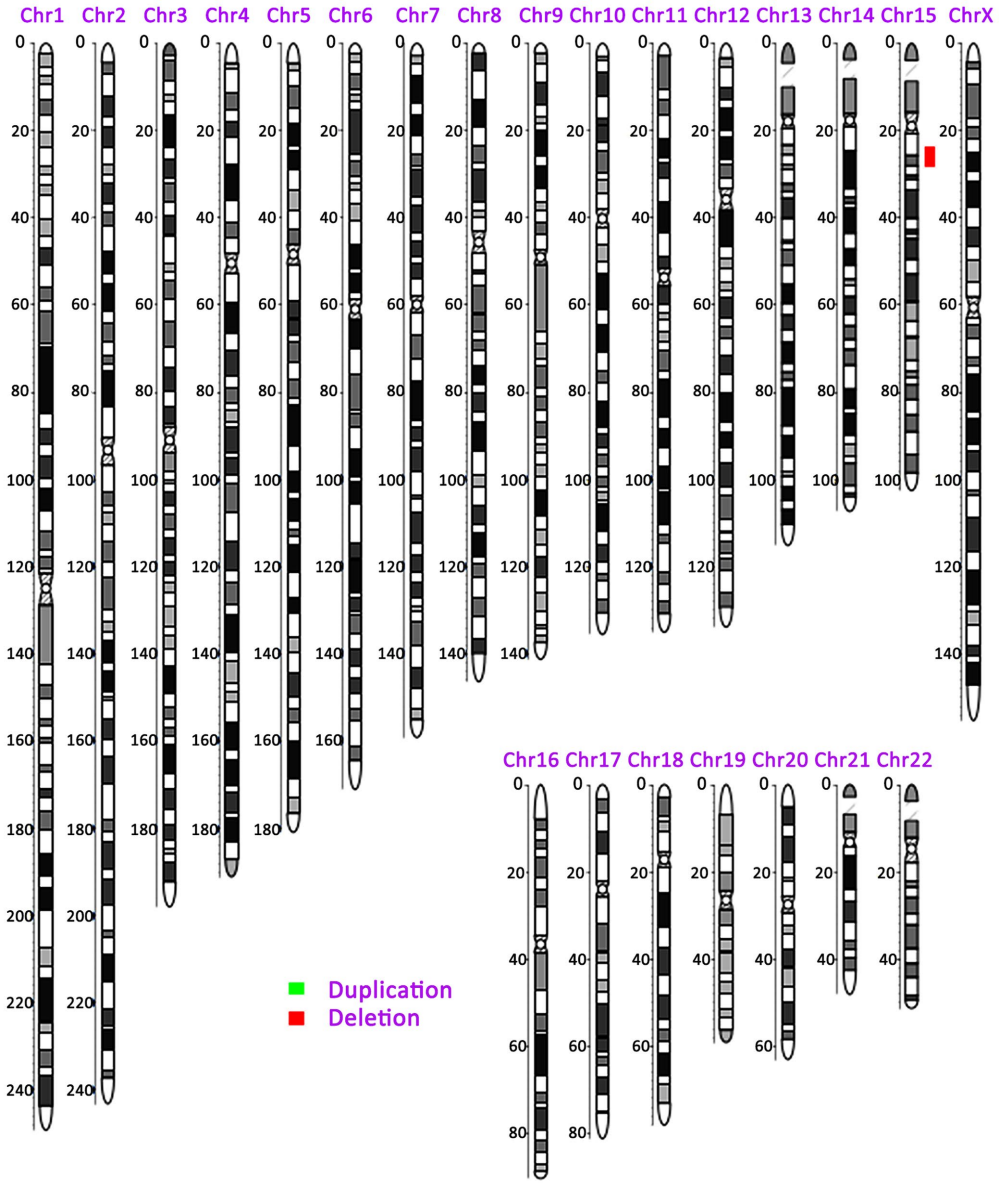
Copy number variation study of maternal amniotic fluid showing that the long arm of fetal chromosome 15 has a deletion of approximately 4.96 Mb (15q11.2‐q13.1; 23.62 Mb‐28.58 Mb)

The CNV analysis results were seq[hg19] 15q11.2‐q13.1 (23.62 Mb‐28.58 Mb)x1, indicating a deletion of about 4.96 Mb on chromosome 15q11.2‐q13.1 (Figure 2). CNV analysis of the chromosomes of both parents and their other children showed no obvious abnormalities.

### 4.3 Karyotype analysis

**Figure 3:**
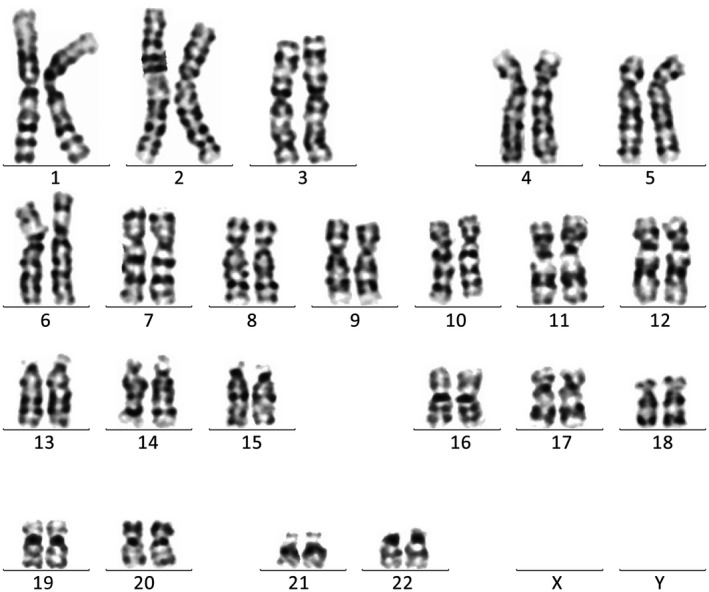
Karyotype analysis of maternal amniotic fluid showing no significant fetal chromosomal abnormalities

Amniotic fluid karyotype analysis showed no obvious abnormalities in fetal chromosome structure (Figure 3).

## 5 DISCUSSION

Fetal chromosomal abnormalities are among the most common types of birth defects seen in clinical practice, with 95% of chromosomal abnormalities being aneuploidies. Common aneuploidies include trisomy 21, trisomy 18, trisomy 13, and sex chromosome abnormalities.^6^, ^7^ Some chromosome abnormalities are due to microdeletions or duplications. At present, the detection techniques for chromosome deletions mainly include karyotype analysis, FISH, and CNV. However, the high cost of detection and the invasiveness of the detection technology make these techniques difficult to use as clinical screening tools, and there is a 1%‐3% risk of abortion in interventional prenatal diagnosis.^8^ NIPT, as a new prenatal screening method, has many advantages, such as being noninvasive, having a high detection rate and a low false‐positive rate, being applicable over a wide range of gestational weeks, requiring less clinical information, being a simple process, and having relatively easy quality control.^9^ NIPT is becoming more accepted by clinicians and patients. Several studies have confirmed that high‐throughput sequencing of fetal‐free DNA in maternal plasma has high detection sensitivity and specificity for noninvasive prenatal testing of common fetal chromosome aneuploidy.^3^, ^10^, ^11^ The detection rates of chromosome 21, 18, and 13 aneuploidies by NIPT are 99.2%, 96.3%, and 91.0%, respectively.^9^ However, there are few reports of detecting chromosome microdeletions and duplications.

Studies found that NIPT technology can detect microdeletions and microduplications greater than 300 Kb in fetal genomes.^12^, ^13^ This study successfully used NIPT to detect microdeletions of about 5 Mb in fetal chromosome 15, which is consistent with the literature. CNV was used to further pinpoint the specific missing regions, confirming the results of NIPT. There was no abnormality observed in the karyotype analysis, which appears to be inconsistent with the NIPT and CNV results. However, identifying chromosome deficiencies and duplications of less than 5 Mb by amniotic fluid karyotype analysis is difficult and they may often be missed.

Most children with chromosomal microdeletions can survive normally, but they may exhibit various degrees of physical or mental developmental abnormalities after birth. The results of the CNV in the study showed that there was a microdeletion of 4.96 Mb in the position of q11.2‐q13.1 (23.62 Mb‐28.58 Mb) of autosomal chromosome 15 in the sample (Figure 1). The deletion of this region of chromosome 15 covers the reported regions of Angel syndrome and Prader‐Willi syndrome (15:23619912‐28438266, about 4.82 Mb), including the pathogenic genes *Ube3a* of Angel syndrome,^14^ and *SNRPN* and *NDN* of Prader‐Willi syndrome.^15^

Angelman syndrome (AS, OMIM 105830) is a neurodevelopmental disorder syndrome, of which 70%‐90% of cases are caused by microdeletion of 15q11‐q13 in the maternal line, 3%‐7% by paternal diploidy (uniparental disomy, UPD) in the 15q11‐q13 region, and 2%‐4% by a single gene defect.^16^ The clinical manifestations are mental retardation, severe speech disorder, facial deformity, secondary cerebellar malformation, ataxia, epilepsy, and abnormal behavior, such as easy‐to‐cause laughter.

Prader‐Willi syndrome (PWS, OMIM 176270) is a multi‐system disease, of which 65%‐75% of cases are caused by microdeletion of 15q11‐q13 in the paternal line, 20%‐30% by maternal diploidy (UPD) in the 15q11‐q13 region, and 1%‐3% by a single gene defect.^17^ The clinical manifestations are growth retardation, difficulty feeding in infancy, obesity due to high appetite and drowsiness in childhood, mental retardation, low muscle tone, short stature, small hands and feet, horseshoe kidney, and genital dysplasia due to hypogonadism.

In the case presented here, the microdeletion mutation in the 15q11.2‐q13.1 region is expected to cause a disease. We verified the parents of the fetus, and the parent's research indicated that the deletion of this region of the fetal chromosome 15 was not inherited from the parents. The chromosomes of their other children had no abnormalities. The abnormality in the present case may be due to the age of the parents, but further research would be needed to establish this.

## 6 CONCLUSION

This report shows that NIPT can detect fetal chromosome 15 abnormalities, including microdeletions. The clinical application of NIPT screening can reduce the number of invasive prenatal diagnoses, reduce the incidence of related iatrogenic abortion, and significantly improve the detection rate of fetal chromosomal abnormalities, including microdeletions, which is more sensitive than karyotype analysis.
